# “You sure feel like you’re alone, kind of flailing away out there”: Family caregiver perspectives of caring for an individual with glioblastoma multiforme

**DOI:** 10.1017/S147895152500015X

**Published:** 2025-02-21

**Authors:** Christy Muasher-Kerwin, Abby Baumbach, Yujun Liu, M. Courtney Hughes

**Affiliations:** 1 Department of Allied Health and Communicative Disorders, Northern Illinois University, Dekalb, IL, USA; 2 Senior Services Associates, Aurora, USA; 3 School of Family and Consumer Sciences, Northern Illinois University, Dekalb, IL, USA; 4 School of Health Studies, Northern Illinois University, Dekalb, IL, USA

**Keywords:** Glioblastoma multiforme, caregiver, rehabilitation services, palliative care, brain cancer

## Abstract

**Objectives:**

Glioblastoma multiforme (GBM) is the most common and aggressive form of brain cancer. Family caregivers of individuals with GBM must navigate a wide range of their care recipients’ physical, cognitive, and psychosocial deficits to provide effective care, which is both mentally and physically demanding. This study aimed to investigate the perspectives of family caregivers of GBM patients about the barriers and challenges they encounter when providing care to their care recipients.

**Methods:**

Nineteen current and former family caregivers for individuals with GBM participated in semi-structured interviews from October 2023 through January 2024. We conducted interviews virtually and used applied thematic analysis to code transcripts to determine themes among participant responses.

**Results:**

Three themes emerged from the interview analysis: (1) overwhelming caregiver burden, (2) difficulties coping with the caregiver role, and (3) gaps in caregiver support. These themes demonstrated a significant physical and mental toll on caregivers and a lack of time for engaging in coping strategies. The family caregivers described a lack of resources, minimal education, and limited time with their medical providers left them feeling ill-prepared for their role. Most family caregivers indicated their care recipients did not use rehabilitation services and the family caregivers expressed confusion about hospice and palliative care.

**Significance of results:**

Family caregivers for individuals with GBM desire more straightforward and proactive information and education about their care recipients from their medical providers. There is an opportunity for more utilization of hospice, palliative, and rehabilitation services to provide necessary training to GBM patients and their caregivers.

## Introduction

Worldwide, there are approximately 250,000 new cases and 200,000 deaths each year due to glioblastoma multiforme (GBM) which is the most aggressive and most common form of brain cancer (Kanderi et al. 2024). In the United States, GBM accounts for 50% of all primary brain tumors (National Brain Tumor Society 2024). GBM most often invades the frontal, temporal, and parietal lobes, leading to deficits in motor function, executive functioning, personality changes, speech production, memory, speech understanding, and sensory awareness. Later in its disease course, GBM typically spreads between multiple lobes of the brain, starting on one side of the brain and crossing to the other hemisphere. As the tumor progresses and occupies more space, increased intracranial pressure leads to brain herniation, which ultimately leads to death (Munakomi and Das 2023; Palmisciano et al. 2022). With an average life expectancy of 14 months and increased dependency in the final stages of life, most GBM patients are candidates for hospice and palliative care services (Giammalva et al. 2018). However, research indicates that GBM patients received palliative care referrals and hospice referrals at 39–40% and 66–76% of the time, respectively (Wu et al. 2021). These pronounced physical, cognitive, and behavioral deficits, the aggressive nature of GBM, and a poor prognosis emphasize the significant burden placed on family caregivers. It is important to note that the existential distress, characterized by feelings of meaninglessness, fear of death, and loss of purpose, often exacerbates the psychological burden faced by caregivers, particularly in demanding contexts. Previous research highlights how existential concerns can deeply influence caregivers’ mental health, impacting their overall well-being and ability to provide care (Applebaum et al. 2016). Family caregivers (“caregivers”) are responsible for the majority of caregiving for brain cancer patients, with most care taking place at home (Pompili et al. 2014). Research involving caregivers of individuals with brain tumors shows that caregivers experience high levels of stress and anxiety (Sherwood et al. 2006). The literature also demonstrates that caregivers for individuals with brain cancer experience worse mental health, a higher caregiver burden, and less time allotted to leisure activities than caregivers for other cancer types (Tallant et al. 2023). Furthermore, brain cancer caregivers reported high levels of frustration when their care recipient exhibited personality changes and a psychological and cognitive decline (Whisenant 2011). These negative experiences among brain cancer caregivers were particularly elevated when social support was low (Reblin et al. 2018). One study found that brain cancer caregivers reported the most difficulty managing the patient’s neurological deficits. The study authors recommended examining sub-groups of brain cancer, such as GBM, to better understand the different needs and distress levels of more debilitating forms of brain cancer (Pointon et al. 2023). Multiple studies have examined caregiving for individuals with GBM specifically (Au et al. 2018; Boele et al. 2023; Flechl et al. 2013). One study focused on the extensive cognitive deficits that can impede a caregiver’s ability to care for their care recipient. Caregivers reported that memory deficits significantly increased the caregiving challenge and substantially interfered with their daily lives (Au et al. 2018). In another study focusing on cognitive dysfunction, poor patient performance was associated with higher caregiver burden, increased caregiver time, and greater difficulty performing caregiver tasks. The literature also shows that individuals caring for someone with a GBM consistently report low quality of life, financial challenges, feelings of burnout, and an overall desire for more information from their medical team (Boele et al. 2023; Flechl et al. 2013). Recent work by Applebaum and colleagues (2024) emphasize that family caregivers of individuals with GBM face an especially demanding role due to the dual burden of managing both oncologic and neurologic symptoms. Caregivers must navigate shifts in family roles, disrupted family dynamics, and emotionally complex communication with medical providers, all while addressing their care recipient’s physical, cognitive, and emotional needs. These challenges make caregiving for GBM patients uniquely complex and emotionally taxing. Overall, limited research focusing specifically on caregivers of individuals with GBM exists. We know that individuals with GBM can have significant cognitive deficits that interfere with caregiving tasks, that their caregivers experience a lower quality of life, and that caregivers would like to have more information from their care recipients’ providers. However, existing research does not indicate how caregivers of individuals with GBM cope with their caregiving role, what training and education they did and did not receive, and what support mechanisms these caregivers would have liked to receive to improve their caregiving experience. This study aims to better understand the challenges and needs of caregivers of individuals with GBM, which can inform future research and program design for interventions for this population.

## Materials and methods

### Data collection

We recruited caregiver participants by distributing a recruitment flyer to national GBM organizations and local hospital groups and encouraging them to share it with their members and GBM patients and caregivers. Participants qualified for the study if they were at least 18 years old and a current primary caregiver to an individual with a GBM or a former caregiver of an individual who died due to a GBM within the last 2 years. We used a semi-structured interview to elicit information from each participant about their experiences. The interview explored the barriers and facilitators caregivers experienced when caring for someone with GBM. See Table 1 for the list of interview questions. One researcher (CM) conducted the interviews via Microsoft Teams or Zoom and recorded each interview using automatic transcription. During each interview, the researcher would ask the participants clarifying questions and reiterate the caregiver’s point of view to ensure an accurate understanding of participant comments. After completion of each interview, this researcher (CM) verified the accuracy of the automatic transcription by reviewing the recording and editing the transcripts as needed. To ensure reflexivity during the interview process, the researcher (CM) maintained an ongoing awareness of potential biases and her influence on the research. Prior to conducting interviews, the researcher engaged in self-reflection to examine assumptions and preconceptions about caregiving experiences. The interview guide included open-ended, neutral questions to encourage participants to share their perspectives without leading their responses. Additionally, the researcher documented reflective notes immediately after each interview to capture her own reactions, thoughts, and any potential biases that could have shaped the interaction. This reflexive approach helped the researcher remain attuned to the participants’ voices while minimizing the impact of her own personal perspectives on the data collection process.

**Table 1.** Interview questions

How has serving as a primary caregiver impacted your own health and well-being? How would you describe your stress levels when providing care? Please describe any cognitive or behavioral changes experienced by the patient for whom you’re caring. How have these changes affected you personally and your ability to provide care? How have you coped with your loved one’s diagnosis and the demands of caregiving? What was the most helpful? What support have you received that has been helpful to you in your role as a caregiver? Who provided that care? For example, your care recipient’s oncologist or a family friend. What training have you received from members of the healthcare team to allow you to care for your loved one? Do you feel that training has been adequate for your needs? Did your loved one receive physical, occupational, and/or speech therapy? What were your experiences with therapy? Is there anything else you would like to share about this topic?

One participant did not have Microsoft Teams or Zoom and provided their responses over the telephone with a second researcher (AB) present during the interview for note taking. We issued each respondent a $50 Amazon gift card for participating in the interview. The Northern Illinois University Institutional Review Board reviewed and approved this study. Each participant provided informed consent prior to taking part in the interview.

### Data analysis

We used applied thematic analysis to identify, analyze, and interpret themes within our data. With this approach, we allowed codes to emerge organically from the data. This was an iterative process where the initial codes evolved as the analysis progressed. We chose thematic analysis to ensure that the caregivers’ voices remained central. We completed interviews until we believed we had sufficient data for thematic saturation based on the concept of theoretical sufficiency. This was confirmed through discussion and identification of consistent threads noted throughout each interview (Morse and Richards 2002). We imported interview transcripts into Dedoose V.9.0.17. The research team independently reviewed the interview transcripts. At least 2 researchers (CM, AB) initially developed codes based on emerging themes within the transcripts via our applied codebook thematic analysis approach (Charmaz 2006). The researchers reviewed each transcript multiple times before developing a code structure. In some instances, they refined the codes to capture the data and identify overarching themes that could be further categorized (Morse and Richards 2002). Any discrepancies in code of theme development were managed via additional transcript reviews and team discussion. The 4-person research team discussed codes, themes, and subthemes to achieve a high level of confirmability (Dey 1999; Hugo and Ganguli 2014).

## Results

**Figure 1.**
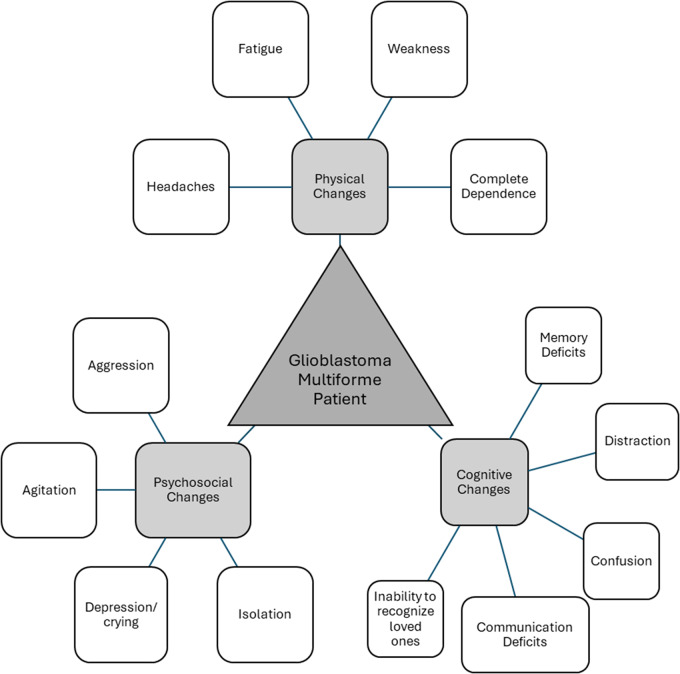
Common types of changes experienced by patients with GBM as described by the participants.

We completed 19 interviews, with interviews lasting 17–55 minutes and, on average, 33 minutes. This number of interviews is just over the 9–17 interviews a systematic review found as a common amount to achieve saturation (Hennink & Kaiser, 2022). The majority (73.7%) of participants were female. The age range was between 25 and 78 years, with an overall average age of 56 and 65–74 being the most represented (31.6%) age-group. Participants were all family members of the care recipients, with the spousal role being the most common (68.0%). There were 11 participants whose care recipients had died and 8 who were currently caring for their care recipient. Most participants (89.5%) lived with the care recipient. Participants were from all major regions across the United States. Table 2 presents detailed participant characteristics. Figure 1 depicts the common care recipient characteristics described by the participants throughout the interviews. We identified 4 key themes in the data and present them below, with illustrative quotes in Table 3.

**Table 2.** Participant characteristics

Demographic profile	Participants, *n* (%)
Gender	
Male	5 (26.3)
Female	14 (73.7)
Age	
25−34	2 (10.5)
35−44	2 (10.5%)
45−54	5 (26.3)
55−64	3 (15.8)
65−74	6 (31.6)
75+	1 (5.3)
Race	
White	19 (100)
Relationship to patient	
Wife	10 (52.6)
Husband	3 (15.8)
Daughter	3 (15.8)
Son	1 (5.3)
Father	1 (5.3)
Sister	1 (5.3)
Care recipient status	
Alive	8 (42.1)
Deceased	11 (57.9)
Living situation	
With care recipient	17 (89.5)
Apart from care recipient	2 (10.5)
Location	
Northeast (PA, NY)	6 (31.6)
Midwest (MI, WI, MN, NE)	5 (26.3)
South (FL, TX, VA)	4 (21.05)
West (CA, NV	4 (21.05)

**Table 3.** Representative quotes from participants

Theme	Quotes (Participant #)
Overwhelming caregiver burden	“So I really couldn’t leave her unattended for any period of time because I was afraid she was going to trip and fall and hurt herself because of that left neglect.” (6) “It’s definitely like having a newborn all over again.” (2) “..you know toward the end I felt like I had already lost my husband and I just transitioned into a caregiver.” (4) “You go from a two-income family … boom … to a one-income family in 30 seconds and trying to keep up with everything and the bills and pay for everything.… It’s just, it’s unbearable.” (3) “It’d be nice to have somebody here all night, but it’s a little bit cost prohibitive to do that.” (12) “There were no doctor appointments. There were no dental appointments. So, my health and wellness essentially went out the window.” (4) “I have hurt my ribs, my elbows, my shoulders.” (5) “Most of the time when I just lose it, it’s in the car, so a lot of it is crying, not so much sleeping.” (13) “I feel like now I feel like I’m his roommate and his caretaker. I don’t feel like his wife.” (8)
Difficulties coping with the caregiver role	“I’m a nurse as well, but you know, the nursing just went out the window when it’s your own spouse.” (3) “You still had to kind of do basically all your research on your own.” (1) “So it’s like even after every appointment, I had no idea what they were talking about and I would be on Google, like Googling these words, ‘cause they don’t, they don’t even have a conversation with you in layman’s terms to tell you what even happened in that visit for the day.” (5) “In a way, I think it’s probably even better than it has been because I’m going to a lot of trouble to make to optimize his health, especially with diet and exercise, which are two things we do together, eating and exercising every day, you know. So, I think it helped us both enormously.” (7) “Coping-wise was just knowing that it wasn’t forever, sadly.” (2) “I would just have to like, leave the room for a few minutes and cry, you know” (4) “Because we really appreciate every good thing in life that we can experience now.” (7) “So I felt if I could at least help somebody who was dealing with something similar, you know, that was positive.” (9) “The caregiver has to be the advocate, they have to really bring their a-game every time with all appointments and be prepared.” (14)
Gaps in caregiver support	“You know like I, I get no help from anyone on his care team when it comes to trying to coordinate appointments, making sure the proper labs are done before he has treatments all of that. If I wouldn’t stay on top of it, no one will and he would fall through the cracks and that’s not abnormal for rural healthcare.” (11) “But with her declining condition I just I never anticipated how quickly would change and I felt I could never get ahead of it and nobody was ever helping me try and anticipate what that was going to look like and where to go for resources and help when it happened.” (6) “The funding isn’t there and the funding needs to be there more so because the treatments for many of these other cancers are focused on drugs that don’t cross the blood brain barrier. But in this case, you need drugs to cross the blood brain barrier.” (9) “So he spent at least two weeks in the hospital having two surgeries in ICU, intubated for 14 days with nobody by his side because they wouldn’t let me there.” (3) “It took us two months to get to a neurologist because COVID was going on.” (3) “I mean, I can’t just talk to her and, you know, tell her how much I love her and how I’m going to miss her and I’ll always be with her. You know, I couldn’t, couldn’t do any of.” (10) “I do have some family, but they live like an hour and a half away. But I wish they were closer to me and geographically so I could lean on them more.” (8) “And when they do say something, it’s often the wrong thing, even though it’s not meant to be, and it changes your relationships.” (9) “The therapist that from the rehab, the inpatient facility were wonderful. They really trained me well and taught me well. And then even the outpatient therapist, when I took him, they were able to give me some hints and some advice on ways to get him in and out of the car easily, more easily.” (3) “Honestly, PT and OT were the best. I still laugh because I remember the first time PT came and they gave us a gait belt. From that day forward, my husband wore that, wore that gait belt 24/7, 24/7.” (4) “I went to occupational therapy again for her vision and I don’t think the person had any clue of how to slow things down because of [my wife’s] processing speed. She just moved way too fast. [My wife] was way overwhelmed. So, I just, I just backed away from it because it was just causing stress.” (6) “And so they would show me how to get him from behind and lift him because we have a lift chair and that’s instrumental in taking care of him, but and they would show us how to transfer him in bed to sitting up. But things in a real setting like this and doing it, it just didn’t work out really that well for us. I mean the PT could do it, you know and stuff, but it was, it was really hard.” (5) “But it was like once PT and OT came in there toward the end, I’m like going, wow, how much easier would some of those things have been earlier on to know?” (1) “Some of the tips and tricks that like occupational therapy, like they were so helpful. It just felt like they were a little late sometimes.” (1) “And after I brought him home, I did take him to a local facility for some physical therapy that only lasted probably a month and he just deteriorated so rapidly that he could no longer do anything.” (3)

### Theme 1: overwhelming caregiver burden

Caregivers reported putting their own lives completely on hold, and they blamed the all-encompassing nature of the GBM patient’s illness. For example, participants stated that their care recipients typically required care every minute of the day, even overnight, with participants often stating the fear of their care recipient falling as a major reason for this vigilance. One participant referred to caring for someone with a GBM as being similar to caring for a newborn baby due to the constant care requirement, fear for their safety, physical dependence, and cognitive challenges. The caregivers reported feeling alone and isolated while making life-altering decisions for their care recipient because GBM prevented their care recipients from understanding information and weighing choices. Feeling isolated was also related to a loss of companionship, with some participants reporting feeling they had transitioned from spouse to full-time caregiver. Participants described experiencing various physical injuries and symptoms and extensive emotional problems such as depression and anxiety while caregiving. Multiple participants experienced injuries to their back, and one participant also cited injuries to her ribs, elbows, and shoulder. Per the participants, these injuries occurred while attempting to physically assist their care recipient with very little training on how to do so. Other participants reported such high levels of depression and anxiety that they required medication and counseling with a mental health professional. Participants also described losing or gaining weight due to stress or an inability to carry out their usual exercise routine. They sometimes stated that such diet and exercise issues left them feeling they had reduced energy to care for their care recipients. Participants expressed significant financial challenges associated with caregiving. They stated concerns about medical expenses and loss of income due to the caregiver or care recipient no longer working. More than half of the caregivers and their care recipients were of working age. Participants also expressed that finances were the main barrier to seeking additional help and support for caregiver respite or uninterrupted sleep at night.

### Theme 2: difficulties coping with the caregiver role

Participants reported difficulties in coping. They often stated that a lack of time was the most significant barrier to using coping strategies. For many, simply leaving the room briefly to cry or take a breath was the most that they could do to manage, given the many needs of their care recipient. Most participants found the best success with coping methods that did not require them to leave home and, hence, their care recipient. These coping methods included educating themselves about GBM, distraction (e.g., gardening, reading, spending time with others, and working), and general support from family, friends, or virtual support groups. Others found that reaching out to help others going through a similar situation was a helpful coping strategy. Additionally, some found that just knowing they were doing everything they could for the care recipient assisted in their coping. A smaller number of participants reported unique caregiver situations for which there are limited resources, such as caring for more than 1 individual at once and caring for a long-term survivor of GBM. A quarter of the participants had jobs in the medical field, which was helpful primarily to enhance their understanding of the diagnosis. However, these participants also acknowledged how different and more difficult it is to care for their care recipients compared to their patients at work.

### Theme 3: gaps in caregiver support

All participants expressed dissatisfaction with the amount of information, education, and support they received from their medical team. They often described feeling ill-prepared to care for their care recipient and stated the following reasons: a lack of resources, minimal education, and limited time with their medical providers. Nearly all participants wanted more knowledge and access to caregiving resources. They suggested caregiver training videos, pamphlets, apps, and how-to books as resources that could help them. A participant from a rural area described an overall lack of resources and medical providers in her area. She expressed having to drive hours to see a neuro-oncologist and feeling a disconnect and lack of collaboration between local providers and the distant neuro-oncology team. She stated that this physical distance created a major barrier to her care recipient receiving adequate care and prevented her from receiving caregiver training. Additionally, multiple participants reported not feeling respected by the medical team and not receiving needed resources. While relating information about their care recipient to medical staff, participants often felt they were not listened to despite their intimate knowledge of the care recipient and their status. Participants also reported minimal direction and prognostic information from the medical team, a lack of clarity about the treatment course, and little preparation and warning about what was to come regarding the health status of the care recipient. Participants whose care recipients had died described feeling completely blindsided by the dramatic physical and cognitive decline that the care recipient experienced, and they expressed that they were not prepared to manage the changes. For example, 1 participant described not realizing that his spouse would eventually be incontinent, need adult diapers, and require total physical assistance to change their diapers and clothing. Aside from emphatically expressing a desire for more information, education, and transparency from their medical team, participants also suggested that having someone to help them problem-solve regarding practical, everyday issues would help them fulfill their caregiving role. For example, 1 participant mentioned that her husband abruptly stopped being able to swallow. She was not sure how to manage this inability to take medications, stay hydrated, and receive nutrition. She wished that she had been able to find solutions in the moment with someone else as those issues arose. One participant suggested that this practical assistance could come in the form of a caregiver network in which caregivers could be paired up to provide emotional and practical support to each other. Participants also expressed difficulties navigating hospice and palliative care. Participants either did not understand the difference between hospice and palliative care or had it implemented too late or not at all. Many stated they wished they had received more education regarding their hospice and palliative care options and more guidance from their medical team. Others stated that the hospice employees, although helpful, were rarely present, so they did not feel adequate support from their hospice team. Multiple participants also reported frustration with the criteria for being accepted into inpatient hospice. In these instances, the medical team deemed that the patient did not qualify for inpatient services, and the care recipient proceeded to pass away a very short time later after much struggle. Three-quarters of the participants mentioned that they either did not have rehabilitation services (physical, occupational, or speech therapy) or that such services came too late in the care recipient’s disease course when the care recipient was limited by severe illness. Most of the small number who did have therapy found it very helpful. However, 1 participant expressed the need for therapists who specialize in neurological therapy to be better equipped to navigate the patient’s complex needs. Participants also expressed they needed more training to reproduce what the therapists could complete with the care recipient.

## Discussion

Our findings showcased the multiplied intensity of caregiving for individuals with GBM, given the disease’s terminal nature and its significant physical, psychosocial, and cognitive impact on patients. We found an overwhelming care burden exists, with caregivers having little time to employ coping strategies. Furthermore, major gaps exist in caregiver support for GBM caregivers. Our study distinguishes itself by addressing the underutilization of rehabilitation, palliative, and hospice care services among patients with GBM and their caregivers, which are areas often overlooked in the existing literature. We build from these gaps in Table 4 where we provide actionable recommendations tailored to clinicians that aim to enhance the accessibility and quality of care for this unique population. Unlike prior research that primarily documents the caregiving burden, our work bridges this evidence with pragmatic solutions, informed by our interviews, to improve both patient and caregiver outcomes. This dual emphasis on identifying systemic deficiencies and proposing targeted interventions ensures our study’s relevance to clinical practice and its contribution to advancing GBM care. Caregivers experienced many negative effects of caregiver burden, specifically due to the provision of 24-hour care, sole-decision making, and financial concerns. These results are consistent with previous research that has shown that patients with brain cancer require significantly more direct care from their caregivers when compared with other care recipients (Au et al. 2022; Sherwood et al. 2006). The emotional challenges experienced by caregivers in our study are also consistent with that of caregivers of individuals with dementia (Hughes et al. 2021). This is not surprising given that both individuals with GBM and dementia suffer from cognitive decline and resulting significant behavioral changes (Acevedo-Vergara et al. 2022; Hugo and Ganguli 2014). Participants in our study reported some relief from their coping strategies, yet sufficient support and respite were still greatly lacking. Similar to previous research, participants utilized problem-focused coping, where the individual proactively and directly acts on the stressor to minimize stress (Ding et al. 2021; Hawken et al. 2018). For example, participants reported that they coped by seeking out their own education about GBM because they did not feel they received sufficient information from their providers. Previous research has shown that brain cancer caregivers consistently feel underprepared for their caregiving role and lack the necessary support and information to provide care effectively (Boele et al. 2023; Chen et al. 2021; Flechl et al. 2013; Paterson et al. 2023; Pointon et al. 2023). Our study demonstrated that caregivers wish they had more access to books, training videos, and caregiver coordinators that could help them problem-solve practical everyday issues. This is consistent with previous research showing the need for more research on skill-building interventions to assist cancer caregivers with coping and providing care (Hughes et al. 2023a). The Illinois Caregiver Assistance and Resource Portal Act is new legislation signed into law to address this need. The resource portal will be a centralized online portal where caregivers in Illinois can find training, educational, and financial resources all in 1 location (Missakian 2024). This study is consistent with past GBM research demonstrating that caregivers also want more clear and direct communication and information about treatment, side effects, and prognosis (Boele et al. 2023; Walsh et al. 2022). Participants in our study often expressed frustration that their neuro-oncologist recommended radiation without explaining its damaging nature and association with lifelong cognitive deficits. Consistent with previous research, participants also wanted the medical community to value their communication and insights but often felt dismissed (Coman et al. 2024). Although research has shown that patients, caregivers, and oncologists have different perceptions of the same conversation, this highlights the need for more consistent and clear communication among patients, caregivers, and healthcare providers (Walsh et al. 2022). Participants expressed some frustrations with the hospice system. Many of these concerns involved limited hospice team support and minimal availability of hands-on care. These findings are similar to previous research showing that hospice employees are helpful but present in a very limited capacity, providing the caregivers minimal respite (McFarlane and Liu 2020; Silveira 2024). Participants were also confused by the difference between hospice and palliative care, a common limitation shown in previous research (Patel et al. 2020). The National Hospice and Palliative Care Organization (NHPCO) and the National Academy for State Health Policy (NASHP) are promoting awareness of hospice and palliative care. The NHPCO’s website *Caring Info* is an example of an initiative to increase public awareness about the difference between and utilization of hospice and palliative care (CaringInfo 2024). This website offers education and valuable information to assist decision-making surrounding serious illness. Such education and awareness may help caregivers better understand the hospice and palliative care role and provide them with more accurate expectations for care. NASHP works with states to educate the public about the role of palliative care and how to access it (Teshale et al. 2021). Some participants in our study reported their patients starting hospice too late in the dying process. Recent research shows that such underutilization of early palliative care use among patients diagnosed with advanced cancer may be attributed to provider or organizational limitations (Hu et al. 2024). A global systematic review on community-based palliative care, a model that integrates care across inpatient and outpatient settings, showed that palliative care initiated in advance of a patient needing to make a final decision about palliative care improved patient and caregiver outcomes. That review also showed that standardized sessions where palliative care team members participated in consistently scheduled team meetings about patients as well as engaging volunteers to assist with palliative care benefited patients and their caregivers (Hughes et al. 2023b). Participants in our study indicated that their care recipients’ treatment and their own caregiving training included little to no physical, occupational, or speech therapy. There is an opportunity to increase the use of rehabilitation services to help GBM patients and their caregivers. A previous study found that only 26% of individuals with GBM received inpatient rehabilitation services following their craniotomy (Roberts et al., 2014). Of the individuals that received inpatient rehabilitation, the vast majority experienced improvements in physical function, cognition, and bladder control. Research has shown that exercise is safe for individuals with brain cancer and can potentially be a predictor of survival for individuals with recurrent glioma (Ruden et al. 2011; Sandler et al. 2021). Research also shows that by providing sufficient training to caregivers and improving their confidence levels, caregivers may reach a higher level of caregiver mastery, contributing to longer survival times for individuals with GBM (Boele et al. 2017). Physical therapy has a primary role in guided exercise prescription and in training and educating patients and caregivers. Physical therapists regularly train caregivers on the safest and most efficient methods of transferring, performing bed mobility, and assisting their care recipient with ambulation. They can also provide valuable education about proper body mechanics and injury avoidance while performing caregiving tasks (Turner et al. 2013; Weir et al. 2022). Furthermore, physical therapists can help GBM patients with pain control, preparing for potential downgrades in function, and equipment training and prescription (Wilson et al. 2017). Occupational therapists also guide GBM patients in strengthening and equipment usage and are experts in training and educating patients and caregivers in activities of daily living (e.g. activities related to personal care such as eating, bathing, and dressing) (Burgess and Jensen 2019). Speech language pathologists have a primary role in addressing deficits in speech, language, cognition, and swallowing for individuals with brain cancer (Mayer 2008). All of these rehabilitation professionals are also active within the hospice setting to assist with end-of-life care (Mueller et al. 2021; Wallace 2013; Wilson et al. 2017). Table 4 presents action steps clinicians, healthcare organizations, and policymakers can take to help improve the experience of family caregivers of individuals with GBM.

**Table 4.** Action steps

Party	Action step
Community-based palliative care team	- Involve interdisciplinary team to provide person-centered care - Conduct consistently scheduled team meetings to discuss patients
Hospice and palliative care professionals	- Educate caregivers regarding the differences and roles of each service - Train caregivers on how to prevent injuries for themselves and their care recipients
Healthcare organizations	- Run caregiver training and support programs - Train volunteers to assist with palliative care
Healthcare professionals in hospitals	- Educate the caregiver about their care recipient’s equipment needs, including what is needed, why it is needed, and how it will be used - Prepare the caregivers for their role upon discharge by explaining the expected frequency and intensity of their caregiving tasks based on their care recipient’s capabilities - Follow up with patients and caregivers after discharge to address any questions and to assist with problem-solving difficulties with daily tasks
Neuro-Oncology team	- Provide prognosis and treatment information directly to patient and caregiver - Refer to other healthcare providers (e.g., hospice & palliative care, rehabilitation services) in a timely manner - Give patient and caregivers resources to prepare caregivers for their role
Policymakers	- Increase legislation to support informal caregivers - Explore strategies to improve access to hospice and palliative care such as expanding coverage, increasing hospice reimbursement rates, and supporting rural providers not traditionally covered for these services
Psychologists	- Provide emotional support surrounding role changes and changes in family dynamics - Offer pre-loss and post-loss grief counseling - Educate caregivers on the importance of and strategies for self-care
Rehabilitation therapists	- Educate and train caregivers to safely assist their care recipients - Train caregivers on how to use equipment - Provide tips and strategies to ease mobility within the home
Social workers	- Assist caregivers in understanding complex medical systems, managing appointments, and communicating effectively with healthcare providers - Identify and connect caregivers with available resources, such as financial assistance programs, respite care, home health services, and community support organizations - Help caregivers create a comprehensive care plan that balances the patient’s needs with the caregiver’s capacity, ensuring continuity of care while minimizing stress

Future research with caregivers of individuals with GBM should focus on evaluating the effectiveness of education programs and policy interventions, such as those outlined in Table 4 of this study. Rigorous studies are needed to assess the impact of these recommendations on caregiver outcomes, such as confidence, emotional well-being, and ability to manage complex caregiving tasks effectively. A recent systematic review of caregiver policies in the United States highlighted the limited academic research examining caregiver policies (Green et al. 2024). Additionally, obtaining updated data on the current utilization of rehabilitation and hospice/palliative care services is essential to identify barriers and underserved populations and inform tailored interventions that address patient and caregiver needs in a dynamic healthcare environment. Exploration of novel caregiver training programs is another critical area for future research. Programs incorporating virtual components, such as telehealth modules and digital resources may be valuable for enhancing caregiver access to training, regardless of geographic or logistical constraints. These initiatives could maximize caregiver confidence and competency, equipping them to better navigate the multifaceted challenges associated with GBM care. Overall, these research directions have the potential to advance the development of evidence-based interventions that optimize caregiver support and improve outcomes GBM patients and their caregivers. Our study had strengths and limitations. One strength was the representation of participants from every major region within the United States. Having similar caregiver perspectives from these different regions indicates that GBM caregiver support and training can be improved upon nationwide. Another strength was that this study targeted GBM specifically as opposed to brain cancer as a whole since GBM has unique characteristics with its aggressive nature and challenging treatment options (Wu et al. 2021). One limitation of the study was the lack of diversity in the demographic distribution of the research participants, with all participants being White. However, it is important to note that research by Ostrom and colleagues using 15 years of national data showed that White individuals accounted for over 80% of GBM cases in the United States (Ostrom et al. 2018). Furthermore, White people tend to have a poorer prognosis when compared to other races (Patel et al. 2019). Another limitation of our study was a higher percentage of female caregiver representation. However, it is valuable to point out that research has shown that females typically outnumber males as caregivers of GBM patients (Au et al. 2022; Boele et al. 2017). Another limitation was the high likelihood of selection bias in that caregivers who chose to participate may have a stronger interest in caregiving, in general, and thus have had different experiences than caregivers who did not choose to participate. Caregiver challenges have been well-documented in the literature. Our results provide an original accounting of these challenges related specifically to caregivers of individuals with GBM and bring greater attention to the underutilization of rehabilitation, palliative, and hospice services for this population. Our study calls for more attention to the caregiver when discussing treatment interventions, resources, and prognosis and providing training for at-home care. Furthermore, our results highlight the need for earlier and more consistent therapy involvement to maximize safety and functional independence for both the individual with GBM and their caregiver. With more collaboration among healthcare providers and a more unified team approach that addresses both the patient and the caregiver, there is potential to improve support for GBM caregivers in this essential role.
